# Analyzing research trends and developments in osseointegration in patients with extremity amputations: Systematic bibliometric analysis and research recommendations

**DOI:** 10.1097/PXR.0000000000000410

**Published:** 2024-11-08

**Authors:** Raphael-Kyrillos M. Saleib, Begüm Pekbay, Michiel H. J. Verhofstad, Maria A. Paping, Mark G. Van Vledder, Oscar J. F. Van Waes

**Affiliations:** 1 Trauma Research Unit, Department of Surgery, Erasmus MC, University Medical Center Rotterdam, Rotterdam, The Netherlands; 2 Osseointegration Center Rotterdam, Rotterdam, The Netherlands; 3 Rijndam Rehabilitation, Rotterdam, The Netherlands

**Keywords:** bone-anchored prostheses, osseointegration, amputees, bibliometric analysis, research trends, scholarly structure

## Abstract

**Background:**

Bone-anchored protheses (BAPs) by means of osseointegrated implants are increasingly being used in amputees with socket-related issues. Clinical advancements are being published by more and more centers worldwide. Although the number of publications and interest in BAP is growing, a systematic evaluation of scholarly output is lacking.

**Objective:**

To identify scholarly output, understand research trends and make research recommendations in the clinical field of BAP.

**Methods:**

Systematic searches in Medline All, Embase, Web of Science Core Collection, Cochrane Library, and Google Scholar were completed in February 2023. The results were deduplicated, screened, and assessed for quality by independent reviewers. Inclusion criteria were as follows: clinical studies and BAP in the extremities. Articles were excluded if they were animal or fundamental studies, nonclinical reports, had a study population less than 10 patients, or BAP was performed in areas other than extremities.

**Results:**

One hundred twelve articles were included and published between 1993 and 2023. An annual growth rate of 10.3% was found and research was published in 62 different journals. Prosthetics and Orthotics International, Clinical Orthopaedics and Related Research and The Bone & Joint Journal were the most prolific journals. *Hagberg K*, *Aschoff HH*, and *Branemark R* were major contributors to BAP research. Collaborations are predominantly among high-income countries. Main research trends were on rehabilitation, questionnaires, complication managements, and implant treatment.

**Conclusions:**

Research on BAP shows an increasing global trend, highlighting key research areas and authors. A unified global research agenda, stakeholders' collaborations, and consensus are essential for addressing knowledge gaps and development future direction of BAP.

## Introduction

The oldest known prosthesis is an ancient Egyptian wooden toe dating back to 1550 – 700 BC.^1^ Advancing to the 16th century, Ambroise Paré designed the first socketed prosthetic (SP) limbs. Despite technical innovations in prosthesis design, the essence of SP limbs has essentially remained the same.^2^ Contemporary limb prostheses still present challenges, such as uncomfortable fits, skin damage, pressure spots, limited energy transfer, and unnatural gait, resulting in reduced activity levels and leading to reduced quality-of-life (QOL), isolation, and nonparticipation.^3–5^ With a growing number of amputations expected, the impact of socket-related problems on patients and the health care system is gaining more attention.^6,7^

By establishing a direct structural and functional connection between the prosthesis and the limb, socket-related issues can be circumvented. After surgery, the implant undergoes a healing phase, leading to the formation of a fibrous capsule layer around the implant. Osteoblasts then deposit bone tissue onto the implant's irregular surface, creating the “osseointegration layer” that securely anchors the implant. This entire biological process is referred to as osseointegration.^8–10^ Bone-anchored prostheses (BAPs), which utilize osseointegrated implants, are increasingly being used in patients with socket-related issues. The growing interest has spurred extensive research and advancements in the field.^11^ The clinical advantages of BAP are also on the rise globally, as more patients and clinics recognize their benefits.^12,13^ These benefits include improved mobility, walking ability, QOL, prosthesis wearing time, (hip) range of motion, reduced oxygen consumption, enhanced energy and force transfers, and better sensory feedback (osseoperception) due to the ground-body interaction through the BAP.^3–5,14–19^

The increasing use of BAPs in clinical settings, along with the rise of various implant systems, has prompted numerous reviews and attempts at meta-analyzing functional and clinical outcomes, often referencing the same research papers.^9,18,20–26^ Although systematic reviews and meta-analyses offer a summary of empirical evidence, a bibliometric analysis delves into influential publications and research trends in the emerging field of BAP, providing academic benefits and fostering collaboration opportunities. The main goal of this study was to present a bibliometric overview, including top-cited journals, authors, articles, and collaborations. Secondary objectives included identifying knowledge gaps and proposing a future research agenda in the field of BAP.

## Methods

To identify the relevant literature in a broad, systematic, and reproducible manner, a systematic bibliometric review was utilized.^27^ Best research practices for conducting a bibliometric study and adopted methodological practices from the Preferred Reporting Items for Systematic reviews and Meta-Analyses statement were applied (Appendix S1, http://links.lww.com/POI/A282).^28–30^

### Data sources

The search was developed in Embase.com, optimized for sensitivity, and then translated to other databases as previously described.^31^ The search was conducted in the databases from inception until February 3, 2023: Medline All, Embase, Web of Science Core Collection, Cochrane Central Register of controlled Trials. Google Scholar was searched for additional articles. The references were imported into EndNote, and duplicates were removed as described previously.^32^

### Search strategy

The search strategies for Embase and Medline used relevant thesaurus terms from Emtree and Medical Subject Headings, respectively. In all databases, terms were searched in titles and abstracts of references. The search contained terms for (1) Osseointegration and (2) Bone-Anchored Prostheses/Prosthesis. Terms were combined with Boolean operators AND and OR, and proximity operators were used to combine terms into phrases. Synonyms and related terms were also used in the search. There were no restrictions on the osseointegration procedure and location of the prosthesis. Animal and fundamental studies were not excluded from the search, nor any language restrictions were made. The searches in Embase and Web of Science were limited to exclude conference papers. The full search strategies of all databases are provided in Appendix S2 (http://links.lww.com/POI/A282). The reference lists of retrieved nonincluded relevant review articles and of the included references have been scanned for relevant references missed by the search. No authors or subject experts were contacted, and unindexed journals in the field were not browsed.

### Selection of studies

Two review authors (“R-KMS and BP”) selected and assessed potentially eligible studies for inclusion in this systematic bibliometric study. Inclusion criteria were as follows: (1) clinical studies and (2) BAP in the extremities. Exclusion criteria were as follows: (1) animal or fundamental studies, (2) nonclinical reports, (3) study population n < 10, and (4) BAP was performed in areas other than extremities. Titles and abstracts of all retrieved references were screened for eligibility by 2 researchers. The content of the full text was analyzed to eliminate articles, which do not meet the inclusion criteria. Decisions by the review authors (“R-KMS and BP”) were documented, and a third review author (“OJFvW”) was available to resolve any disagreement. Discrepancies were resolved through discussion at each stage.

### Bibliometric toolbox and analysis

The open-source bibliometric software *bibliometrix* was utilized, which is a tool for quantitative research in scientometrics that includes the main bibliometric methods for data extraction from databases and analysis.^33^ Metadata were retrieved from Clarivate and the PubMed API. Both performance analysis and science mapping from the bibliometric analysis technique toolbox were used.^28^ In the performance analysis, the number of locally and globally cited authors, publishing journals, and published articles were ranked. Note, that *local* depicts the frequency of included journals, authors, and articles of the selected articles in this bibliometric review and that *global* depicts the citation of the journals, authors, and articles in the references of the included articles in this review. Performance was also expressed by the Hirsch-index (*h*-index). The *h*-index is defined as the maximum value of *h* such that the given author and journal has published at least *h* papers that have each been cited at least *h* times.^34^ For the science mapping, collaboration between countries, that is articles that have (co-)authorships in different countries, was visualized. In addition, thematic keyword mapping by plotting the development degree (density) over the relevancy degree (centrality) of the keywords was performed as part of this analysis.

### Medical ethical approval

As this study is a review and does not involve patients, medical ethical approval was not required in the Netherlands.

## Results

### Search strategy

**Figure 1.**
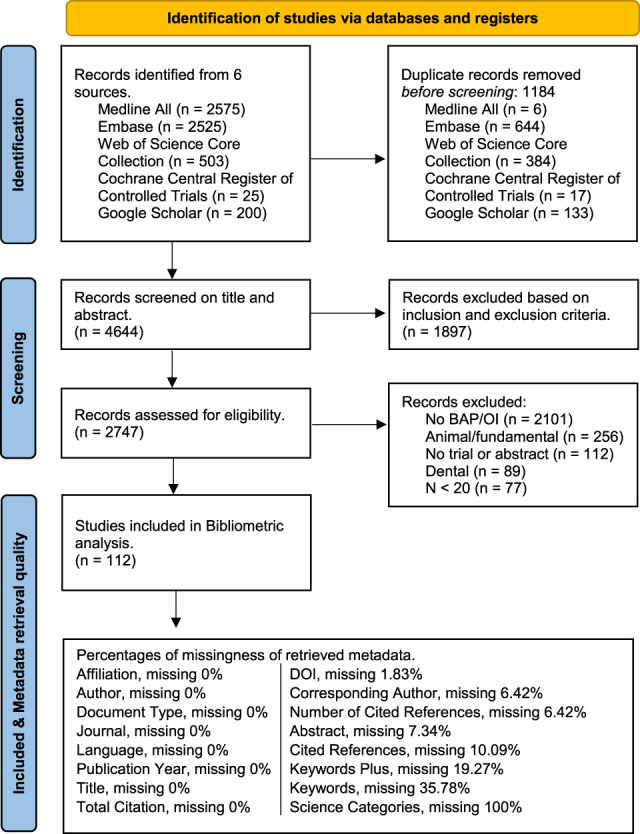
Flowchart of included studies and data retrieval quality. Abbreviations: BAP, bone-anchored protheses; DOI, digital object identifier; OI, osseointegration.

A total of 5828 articles were retrieved. After deduplication, 4644 articles were screened on title and/or abstract. Of these, 2747 articles underwent full-text screening. And 2638 articles were excluded for various reasons (Figure 1), leaving 112 articles included for metadata analysis (Appendix S3 for references, http://links.lww.com/POI/A282).

### Scientific production

**Figure 2.**
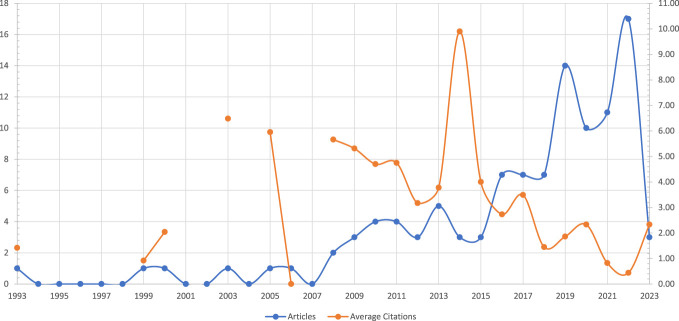
Articles and Average Citations per year. Note that the x-axis on the left depicts the number of articles (in blue) published in that year and that the scale of the right axis differs and depicts the number of average citations (in orange) for given year. A year with no publications does also not have an average citation for that year.

The studies spanned from 1993 to 2023, showing an annual growth rate of 10.3% (Table 1). A total of 281 unique (co-)authors were identified, averaging 4.5 coauthors per article. The annual volume of new articles and average citations are depicted in Figure 2. The year 2022 recorded the highest number of published articles (n=18).

**Table 1.**
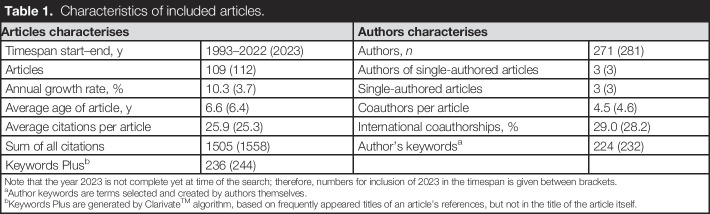
Characteristics of included articles.

Articles characterises		Authors characterises	
Timespan start–end, y	1993–2022 (2023)	Authors, *n*	271 (281)
Articles	109 (112)	Authors of single-authored articles	3 (3)
Annual growth rate, %	10.3 (3.7)	Single-authored articles	3 (3)
Average age of article, y	6.6 (6.4)	Coauthors per article	4.5 (4.6)
Average citations per article	25.9 (25.3)	International coauthorships, %	29.0 (28.2)
Sum of all citations	1505 (1558)	Author's keywords^a^	224 (232)
Keywords Plus^b^	236 (244)		

Note that the year 2023 is not complete yet at time of the search; therefore, numbers for inclusion of 2023 in the timespan is given between brackets.

a Author keywords are terms selected and created by authors themselves.

b Keywords Plus are generated by Clarivate^TM^ algorithm, based on frequently appeared titles of an article's references, but not in the title of the article itself.

### Top cited journals

Osseointegration research was featured in 62 journals. *Prosthetics and Orthotics International* has the highest impact on scholarly output with 15 published articles, 308 citations, and an *h*-index of 11 (Table 2). *Clinical Orthopaedics and Related Research* and *The Bone & Joint Journal* came in second and third with 6 and 5 articles included in this study, respectively. The *Journal of Rehabilitation Research & Development* and the *Archives of Physical Medicine and Rehabilitation* were the second and third most cited journals with 226 and 164 cited articles, respectively. *The Bone & Joint Journal* had the second highest h-index.

**Table 2.**
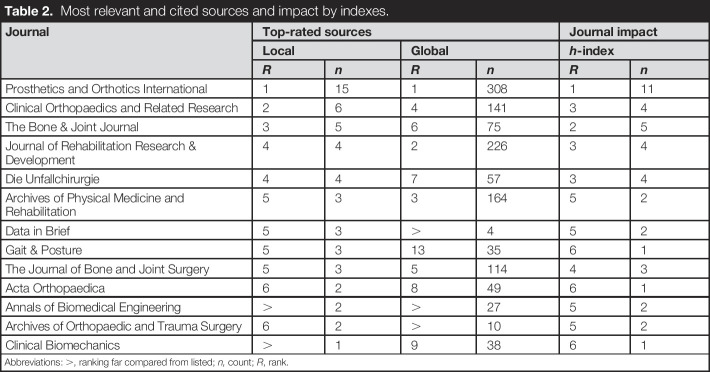
Most relevant and cited sources and impact by indexes.

Journal	Top-rated sources				Journal impact	
	Local		Global		*h*-index	
	*R*	*n*	*R*	*n*	*R*	*n*
Prosthetics and Orthotics International	1	15	1	308	1	11
Clinical Orthopaedics and Related Research	2	6	4	141	3	4
The Bone & Joint Journal	3	5	6	75	2	5
Journal of Rehabilitation Research & Development	4	4	2	226	3	4
Die Unfallchirurgie	4	4	7	57	3	4
Archives of Physical Medicine and Rehabilitation	5	3	3	164	5	2
Data in Brief	5	3	>	4	5	2
Gait & Posture	5	3	13	35	6	1
The Journal of Bone and Joint Surgery	5	3	5	114	4	3
Acta Orthopaedica	6	2	8	49	6	1
Annals of Biomedical Engineering	>	2	>	27	5	2
Archives of Orthopaedic and Trauma Surgery	6	2	>	10	5	2
Clinical Biomechanics	>	1	9	38	6	1

Abbreviations: >, ranking far compared from listed; *n,* count; *R*, rank.

### Top cited authors and Worldwide collaborations

**Figure 3.**
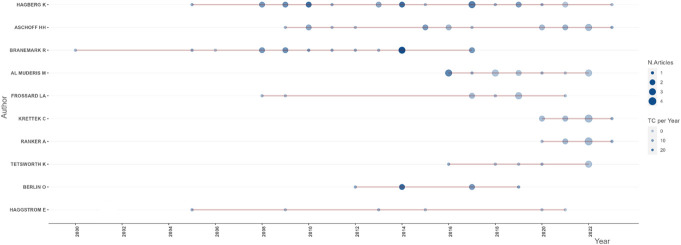
Authors production over time. Authors are displayed on the x-axis and years on the y-axis. The bubble size is proportional to the number of articles, whereas the color intensity in blue is proportional to the total citations per year. The line represents the timeline of given author. Abbreviations: N, number; TC, total citation.

**Figure 4.**
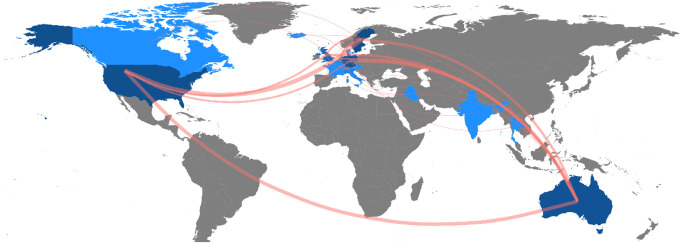
World map showing global research collaborations.

Osseointegration research was published by 281 authors. The author with the highest number of published articles was *Hagberg K*, with 24 articles (co-)authored, 240 global citations, and *h*-index of 18 (Table 3). *Aschoff HH* (n = 18) and *Branemark R* (n = 17) were the second and third most prolific authors, respectively. *Branemark R* was the second highest cited author (235) and *h*-index (16). The 3 mentioned authors also have the longest continuing production of articles over time (Figure 3). As authors are affiliated with institutions and bound by geographic locations, research collaboration from a worldwide perspective can be mapped. A world map indicates that collaborations in osseointegration research are predominantly among high-income countries (Figure 4).

**Table 3.**
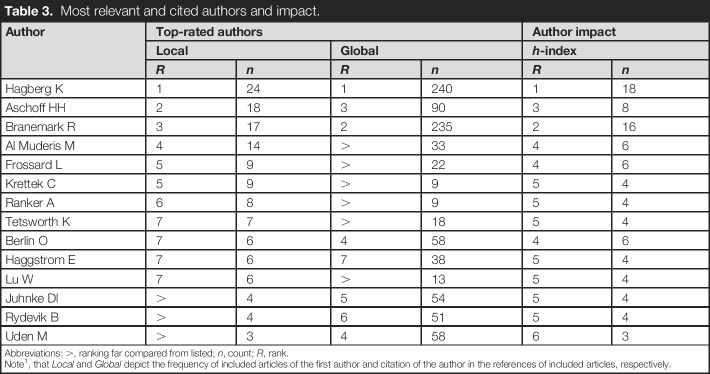
Most relevant and cited authors and impact.

Author	Top-rated authors				Author impact	
	Local		Global		*h*-index	
	*R*	*n*	*R*	*n*	*R*	*n*
Hagberg K	1	24	1	240	1	18
Aschoff HH	2	18	3	90	3	8
Branemark R	3	17	2	235	2	16
Al Muderis M	4	14	>	33	4	6
Frossard L	5	9	>	22	4	6
Krettek C	5	9	>	9	5	4
Ranker A	6	8	>	9	5	4
Tetsworth K	7	7	>	18	5	4
Berlin O	7	6	4	58	4	6
Haggstrom E	7	6	7	38	5	4
Lu W	7	6	>	13	5	4
Juhnke Dl	>	4	5	54	5	4
Rydevik B	>	4	6	51	5	4
Uden M	>	3	4	58	6	3

Abbreviations: >, ranking far compared from listed; *n*, count; *R*, rank.

Note^1^, that *Local* and *Global* depict the frequency of included articles of the first author and citation of the author in the references of included articles, respectively.

### Top cited articles

The article, “One hundred patients treated with osseointegrated transfemoral amputation prostheses—Rehabilitation perspective” leads in citation, with 43 local and 170 global citations (Table 4).^35^ Second was “*A novel osseointegrated percutaneous prosthetic system for the treatment of patients with transfemoral amputation*,” with 37 local citations and 157 global citations.^36^ The third, “*Osseointegrated Titanium Implants for Limb Prostheses Attachments: Infectious Complications*,” had 151 global citations.^37^ All of the top cited articles focused on lower extremity amputees.

**Table 4.**
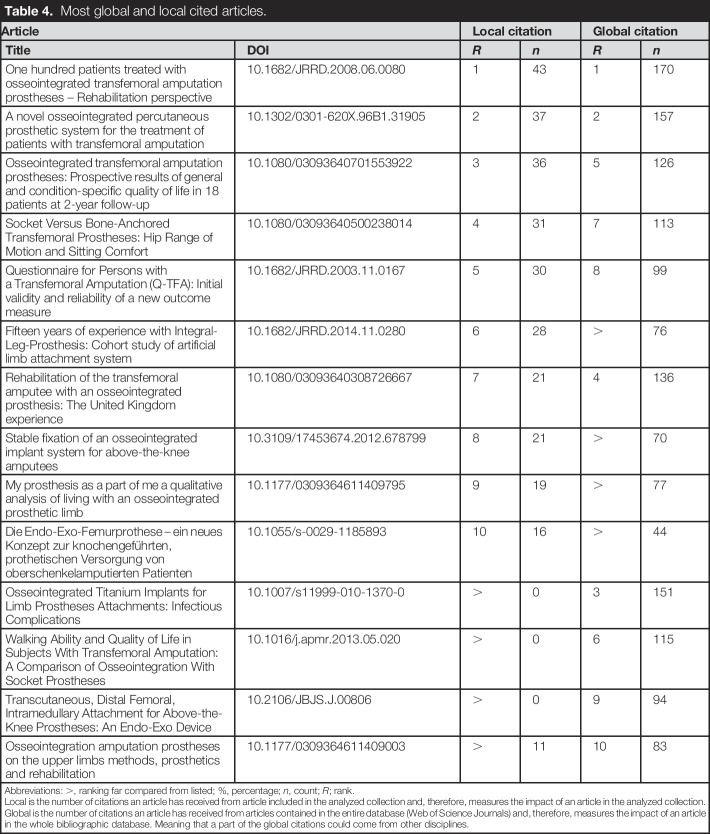
Most global and local cited articles.

Article		Local citation		Global citation	
Title	DOI	*R*	*n*	*R*	*n*
One hundred patients treated with osseointegrated transfemoral amputation prostheses – Rehabilitation perspective	10.1682/JRRD.2008.06.0080	1	43	1	170
A novel osseointegrated percutaneous prosthetic system for the treatment of patients with transfemoral amputation	10.1302/0301-620X.96B1.31905	2	37	2	157
Osseointegrated transfemoral amputation prostheses: Prospective results of general and condition-specific quality of life in 18 patients at 2-year follow-up	10.1080/03093640701553922	3	36	5	126
Socket Versus Bone-Anchored Transfemoral Prostheses: Hip Range of Motion and Sitting Comfort	10.1080/03093640500238014	4	31	7	113
Questionnaire for Persons with a Transfemoral Amputation (Q-TFA): Initial validity and reliability of a new outcome measure	10.1682/JRRD.2003.11.0167	5	30	8	99
Fifteen years of experience with Integral-Leg-Prosthesis: Cohort study of artificial limb attachment system	10.1682/JRRD.2014.11.0280	6	28	>	76
Rehabilitation of the transfemoral amputee with an osseointegrated prosthesis: The United Kingdom experience	10.1080/03093640308726667	7	21	4	136
Stable fixation of an osseointegrated implant system for above-the-knee amputees	10.3109/17453674.2012.678799	8	21	>	70
My prosthesis as a part of me a qualitative analysis of living with an osseointegrated prosthetic limb	10.1177/0309364611409795	9	19	>	77
Die Endo-Exo-Femurprothese – ein neues Konzept zur knochengeführten, prothetischen Versorgung von oberschenkelamputierten Patienten	10.1055/s-0029-1185893	10	16	>	44
Osseointegrated Titanium Implants for Limb Prostheses Attachments: Infectious Complications	10.1007/s11999-010-1370-0	>	0	3	151
Walking Ability and Quality of Life in Subjects With Transfemoral Amputation: A Comparison of Osseointegration With Socket Prostheses	10.1016/j.apmr.2013.05.020	>	0	6	115
Transcutaneous, Distal Femoral, Intramedullary Attachment for Above-the-Knee Prostheses: An Endo-Exo Device	10.2106/JBJS.J.00806	>	0	9	94
Osseointegration amputation prostheses on the upper limbs methods, prosthetics and rehabilitation	10.1177/0309364611409003	>	11	10	83

Abbreviations: >, ranking far compared from listed; %, percentage; *n*, count; *R*; rank.

Local is the number of citations an article has received from article included in the analyzed collection and, therefore, measures the impact of an article in the analyzed collection. Global is the number of citations an article has received from articles contained in the entire database (Web of Science Journals) and, therefore, measures the impact of an article in the whole bibliographic database. Meaning that a part of the global citations could come from other disciplines.

### Keywords and themes

**Figure 5.**
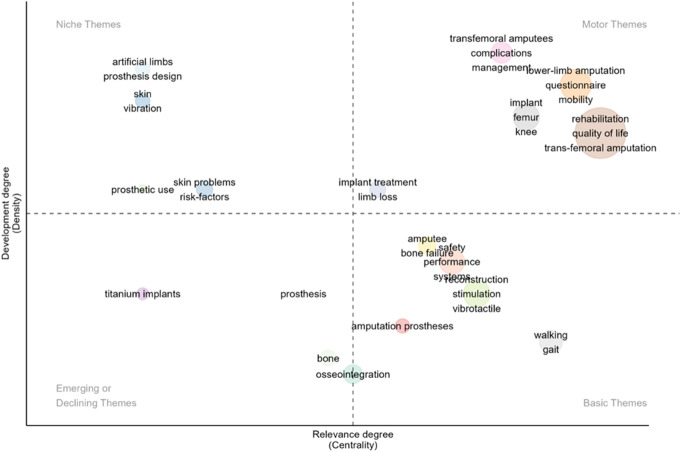
Themes in osseointegration research.

The 3 most frequently used keywords by authors were “*osseointegration*,” “*amputation*,” and “*transfemoral amputation*” occurring 58, 32, and 25 times, respectively (Table 5). Using the KeyWords Plus algorithm, which identifies keywords appearing in the titles of an article's references but not in the article's title itself, we found different trends. The most frequent keywords were “*rehabilitation*” (40 occurrences), “*quality of life*” (37 occurrences), and “*transfemoral amputation*” (also 37 occurrences). In terms of main research themes, many papers focused on “*rehabilitation*,” “*questionnaire*,” “*complications management*,” and “*implant treatment*” (Figure 5). “*Gait*” was identified as a basic research theme in this field. Niche themes included “*prosthetic use*,” “*skin problems*,” and “*vibration*” in BAP research. Meanwhile, “*titanium implants*” and “*bone*” were emerging themes.

**Table 5.**
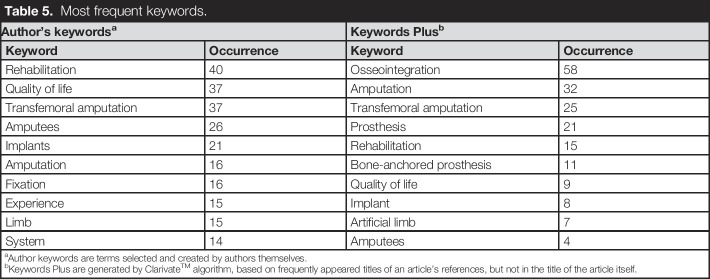
Most frequent keywords.

Author's keywords^a^		Keywords Plus^b^	
Keyword	Occurrence	Keyword	Occurrence
Rehabilitation	40	Osseointegration	58
Quality of life	37	Amputation	32
Transfemoral amputation	37	Transfemoral amputation	25
Amputees	26	Prosthesis	21
Implants	21	Rehabilitation	15
Amputation	16	Bone-anchored prosthesis	11
Fixation	16	Quality of life	9
Experience	15	Implant	8
Limb	15	Artificial limb	7
System	14	Amputees	4

a Author keywords are terms selected and created by authors themselves.

b Keywords Plus are generated by Clarivate^TM^ algorithm, based on frequently appeared titles of an article's references, but not in the title of the article itself.

## Discussion

The clinical benefits of BAP for amputees with socket-related issues are increasingly acknowledged worldwide. In recent years, multiple centers have published new clinical findings on this topic, reflecting growing interest. This bibliometric analysis, a first in this field, provides a comprehensive overview of influential publications and research. A notable exponential growth in BAP research, originating primarily from high-income countries, is evident, with these findings frequently published in top journals and involving international collaborations. The most cited paper “One hundred patients treated with osseointegrated transfemoral amputation prostheses—rehabilitation perspective” reports on a rehabilitation protocol after the (two-stage) surgical procedure.^35^ And the second most cited, “*A novel osseointegrated percutaneous prosthetic system for the treatment of patients with transfemoral amputation*” report on the 24 months positive changes of health survey questions between baseline and the 2-year 92% cumulative implant survival.^36^ Interesting topics are also found, in the bibliometric analysis, to be main themes too: “*rehabilitation*,” “*questionnaire*,” “*complications management*,” and “*implant treatment*.” Note that the most important articles were related to lower extremity amputations. The results of the bibliometric review, especially the theme topics and research trends, are interesting to explore in more detail, and some research recommendations are made here below.

### Rehabilitation

Regarding rehabilitation, there is an emerging focus on optimizing the rehabilitation pathway. Literature highlights key aspects of rehabilitation time, program, progress, and process.^8,14,35,38–42^ Notably, research from Colorado, USA, emphasizes patient-centered rehabilitation and understanding the biomechanics of human-implant interaction. This includes observations on reduced joint overload and improved walking gait postsurgery.^43^ Colleges from Australia and the United States have also shed light on the anthropomorphicity of prosthetic feet fitted to bone-anchored transtibial prostheses and provided data and experience.^44,45^

Functional tests like the Time Up and Go test and 6-Minute Walking Test are commonly used to evaluate mobility and walking ability. Although balance is inherently part of walking and thus these functional outcomes, research on losing balance, i.e., tripping and falling mechanics, is not measured. Objectively measuring this, and other gait analytics is needed. An example is a study on the activity of daily living showed clinically and statistically significant improvement up to 24 months through an activity monitor for BAP users.^19^ Many stakeholders are involved in the rehabilitation process, and we would like to emphasize the role of prosthetist and recommendations described previously (ISPO and WHO standards for prosthetics and orthotics) in BAP research.

Finally, some examples of objective measurements are through a goniometer,^46^ by three-dimensional gait analysis,^47^ by transducer measuring temporal gait properties,^48^ and on activity limitations by oxygen consumption.^49^

### Questionnaires

Questionnaires, notably *The Questionnaire for Persons with Transfemoral Amputation* (Q-TFA) and The Short-Form 36 Health Survey, are widely used as patient-reported outcomes to evaluate progress in BAP-related studies.^9,18,22,25,26^ It is recommended to establish consensus on which domains should be measured through whatsoever instrument (questionnaire, functional or objective outcome, etc.) by a framework, as recommended previously.^50^

Health Economic Evaluations (HEE) often employ questionnaires to compare costs and cost-effectiveness between standard care and BAP treatments. The outcome of HEE studies are highly dependent on factors such as perspective, time-horizon, healthcare system, and reimbursement structure and need to be updated over time to remain relevant.^51^ Haggstrom et al^52^ demonstrated comparable prosthetic costs and service between BAP and SP, but BAPs cost more due to the use of advanced prosthetic components. It is anticipated that costs will decrease over time due to market forces and optimization. Frossard et al showed that BAP vs. SP was cost-saving and cost-effective for 19% and 88% of the participants, respectively.^53^ Other cost-effectiveness studies from the United Kingdom and United States concluded that there are both QOL and financial arguments in favor of BAP, and that it offers a higher QOL at affordable costs compared with poorly tolerated SP users.^54,55^

In HEE, disease burden is measured to assess the value of medical interventions. BAP centers are encouraged to report the disease burden of their patients' populations. Preferably by the country specific EuroQol-5 Dimension (EQ-5D) Index,^56^ and convert their QOL questionnaires onto the EQ-5D.^57,58^ Furthermore, to fully capture the value of BAP, use the EQ-5D in short-, middle-, and long-term evaluation, particularly before BAP in patients with socket-related issues eligible and ineligible for implantation, at least twice before osseointegration surgery.

HEE should encompass a broad range of costs, including healthcare consumption and productivity losses.^59,60^ The patient's perspective on the received care and its quality is also important to report. These Patient-Reported Experience Measures can impact the optimization of BAP therapy and guidelines. Stated preferences, captured through methodologies like discrete choice experiments, are also vital in understanding patient preferences and patient centered informed shared decision making.^61,62^ A recent study utilizing discrete choice experiments revealed what patients' preferences for BAP matter and can used by policy makers and clincians to understand patients' perspectives, manage patients' expectations, determine associated losses in QOL points and monetary loss secondary to complications.^63^

### Complication management

Complications are a critical focus, as evidenced by the most cited articles in our review. Al Muderis et al^64^ developed a classification system to grade infections based on clinical and radiographic findings. We recommend the unification and reporting of infections and encourage to utilize, enhance, or propose alternative systems that encompass a broader range of adverse events and complications. A systematic review by Atallah et al^24^ emphasizes the need for an international database or a standardized set of complications, along with clear classification systems.

### Implant treatment

Osseointegration surgery can be performed in a single- and two-stage procedure. The rationale behind a two-stage surgery is to provide a sterile period for osseointegration, a practice that has been common since the introduction of the press-fit system in 1999 (Lübeck, Germany),^65^ and has proven to be safe.^24^ However, in 2014, a single-stage procedure was introduced in Australia as standard care.^38^ Different centers prefer the single-stage procedure due to its inherent lower risk of surgical site infections compared with 2 surgeries. Direct comparative studies between these techniques are lacking, and their relative safety remains to be established. However a recent systematic review comparing indirectely the stage procedures has been published recently and suggested that the one-stage approach is favourable compared to the two-stage.^66^

Implant fixation checking could possibly be performed through radiostereometric analysis (RSA), which was done for the Osseointegrated Prostheses for the Rehabilitation of Amputees, Integrum AB, Mölndal, Sweden system by Nebergall, Audrey et al.^67^ A more patient-friendly approach could be a model-based RSA, which avoids the need for inserting tantalum beads. The outcomes from a model-based RSA highlight the importance of cautious monitoring due to complication risks (10 implant removals out of 17 within 5 years).^68^

### Collaboration

Although some recommendations are already mentioned above, 1 more recommendation is made regarding working together. The need for collaboration is essential and can take various forms. Effective collaboration could include establishing international databases for reporting, standardizing reporting items and classification systems, and exchanging knowledge and expertise through collaborative meetings or societies. An example of such collaboration is the Special Interest Group for Bone Anchored Limbs, presented during the ISPO World Congress 2023 (https://www.ispoint.org/special-interest-groups/bone-anchored-limbs). Traditional research methods, like the Delphi technique, could be conducted to find global consensus.

### Limitations

This study has several limitations. First, our search was limited to database-provided metadata, potentially overlooking factors like paper quality or patient population overlap, which require manual article review. Second, our focus on clinical articles may have excluded fundamental research. In addition, metadata can change over time postpublication, affecting citation counts or other details. Finally, variability in terminology and naming conventions in the metadata could introduce bias.

## Conclusion

Research on BAP for amputees shows an increasing global trend, highlighting key research areas and authors. A unified global research agenda, stakeholders' collaborations, and consensus are essential for addressing knowledge gaps and guiding future research.

## Funding

The author(s) disclosed that they received no financial support for the research, authorship, and/or publication of this article.

## Declaration of conflicting interest

The author(s) disclosed no potential conflicts of interest with respect to the research, authorship, and/or publication of this article.

## Supplemental material

No supplemental digital content is available in this article.

## Supplementary Material

SUPPLEMENTARY MATERIAL
